# Retention of Urine from Stricture--Perineal Section by A. J. Baxter, M.D., Chicago--Recovery

**Published:** 1867-10

**Authors:** P. Curran


### RETENTION OF URINE FROM STRICTURE--PERINEAL SECTION BY A. J. BAXTER, M.D., CHICAGO--RECOVERY.

Reported by P. CURRAN, M.D.

George B., *æt.* 35, residing in Chicago, a robust man, of an apparently good constitution, states that 17 years ago he had clap; then used strong injections, and about a year and a-half afterwards felt annoyance from stricture; was treated with bougies with benefit. He had clap several times since.

In the spring of 1867, the stricture became so closed that there was almost an impossibility of passing the least urine. He was 24 hours in this condition, when his state became dangerous and, to him, intolerable. The necessity of an operation being urgent, Dr. MacDonald, his physician, referred him to Dr. Baxter, for the purpose of having the operation of perineal section performed. All agreed as to its necessity. Dr. Baxter, accompanied by Prof. Freer, undertook to perform Syme's operation, as the only thing to be done under the circumstances. Dr. MacDonald administered chloroform, and the writer witnessed the operation. The principal difficulty was the impossibility of passing even the smallest bougie, which might serve for a guide. However, a sound was passed into the urethra, as far as it would go, and then Dr. B. cut through the perineum until he touched the end of the sound, thence he advanced the scalpel along the urethra, being followed by the sound, until he cut through the seat of the stricture. This required no small degree of skill, as he had nothing to guide him but his judgment. The insertion of a bougie through the wound determined the success of the operation. A catheter relieved the bladder, and rescued the man from imminent danger. The patient recovered very rapidly, and is now in perfect health. A No. 8 bougie passes through the former seat of stricture with comparative ease.

